# Effects of Reciproc, Mtwo and ProTaper Instruments on Formation of Root Fracture

**DOI:** 10.7508/iej.2015.04.009

**Published:** 2015

**Authors:** Sahar Jalali, Behrooz Eftekhar, Payam Paymanpour, Mohammad Yazdizadeh, Mansour Jafarzadeh

**Affiliations:** a *Department of Endodontics, Dental School, Ahvaz Jundishapur University of Medical Sciences, Ahvaz, Iran; *; b * Department of Endodontics, Dental School, Shahid Beheshti University of Medical Sciences, Tehran, Iran*

**Keywords:** Cracks, Craze Lines, Dentin, Nickel-Titanium Instruments, Root Canal Preparation, Tooth Root, Vertical Root Fracture

## Abstract

**Introduction:**

The aim of this study was to compare the formation of dentinal crack and craze lines in the root dentin during root canal preparation with three different NiTi endodontic systems, naming Reciproc (RCP), ProTaper Universal (PTU) and Mtwo.

**Methods and Materials:**

One hundred extracted mandibular premolars with single canals were selected and decoronated. The teeth were randomly divided into four groups of 25 each (*n*=25). In groups 1, 2 and 3 the teeth were prepared using Mtwo, PTU and RCP, respectively. While in group 4 (control group) the samples were left unprepared. After preparation, all specimens were sectioned perpendicular to the long axis of root at 3, 5 and 9-mm distances from the apex. The sections were then individually observed under 12× magnification using stereomicroscope. The data was analyzed using the chi-square and Fisher’s exact tests. The level of significance was set at 0.05.

**Results:**

No cracks were observed in the control group. All engine-driven systems caused dentinal cracks. Mtwo and PTU caused cracks significantly more than RCP (*P*<0.05). There was no significant difference between RCP and control group (*P*>0.05).

**Conclusion:**

All three engine-driven systems created dentinal defects. Reciproc caused less cracks than Mtwo and ProTaper Universal.

## Introduction

Using NiTi engine-driven instruments for root canal preparation has become the fundamental of endodontic treatments. These instruments have many advantages such as less operation time, increased cleanliness of root canal walls and fewer procedural accidents (apical canal transportation, perforations and ledges) [1]. These properties mostly stem from the increased flexibility of NiTi alloy which helps in preservation of root canal curvatures [2]. However, it is stated that engine-driven instruments may damage root dentin by forming craze lines and microcracks [1, 3]. During root canal preparation, thinned dentinal walls and increased strain can lead to microcrack formation especially at the apical area [4, 5].

These defects might propagate and proceed into greater fractures or vertical root fracture (VRF). VRF is the cause of 10.9 to 31% of tooth extractions [6]. There is a direct relationship between the amount of dentin removal and crack formation; the greater the canal enlargement, the higher the incidence of VRF [3]. On the other hand, cleanliness of prepared canal walls depends on removal of surrounding infected dentin [4]. There is still a dilemma in degree of root canal enlargement and apical preparation to reach the least possible level of bacterial counts. 

ProTaper (Dentsply Maillefer, Ballaigues, Switzerland) is amongst the pioneer engine-driven instruments that employs full 360^°^ rotation with a convex triangular cross-section and multiple tapers within the shaft. The ProTaper Universal (PTU) system is comprised of shaping (SX, S1 and S2) and finishing (F1, F2, F3) instruments [7].

Mtwo rotary files (VDW, Munich, Germany) have an S-shaped cross-section with deep cutting edges and low radial contact that increase the instrument flexibility and improve file performance [8]. Mtwo provides small-sized files (10/0.04 and 15/0.05) that enable reaching the apical third at the beginning of canal preparation by keeping the canal walls unchanged [9].

Newer generations of engine-driven systems may create less damage to root canal walls [1]. Changes in the shape, design of the instruments and type of motion (*i.e. *reciprocation instead of rotation), seem promising. In reciprocation motion, file moves toward apical region by itself with no need to exert more apical force [10]. This kind of movement also reduces cyclic fatigue of instrument more than rotation [11]. Reciproc (RCP) (VDW, Munich, Germany) is one of the new single-file systems working with reciprocating movement. With S-shaped cross-section and a non-cutting tip, this single file shapes the canal by 150 degrees counterclockwise and then 30 degrees clockwise motion with a speed of 300 rpm. This single file system offers three different sizes [R25 (25/0.08), R40 (40/0.06) and R50 (50/0.05)] [7, 12]. 

The aim of this study was to compare the formation of dentinal defect after root canal preparation with three mentioned systems (PTU, Mtwo and RCP).

## Materials and Methods

This study was conducted in department of endodontics, Ahvaz, Iran. One hundred extracted human single-rooted mandibular premolars were selected for this study. The teeth had been extracted for periodontal/orthodontic reasons and were stored in purified distilled water throughout the study. Radiographic evaluation was performed to exclude presence of resorption defects and root canal obliteration. Root canal curvature was 20-30^°^ according to the method introduced by Schneider [13]. Radii of curvature ranged between 5.2 and 10.1 mm.

Teeth were examined to confirm the absence of cracks/fractures under a stereomicroscope at 3× magnification (Zeiss, SV6, Jena, Germany) [14]. The teeth were decoronated at CEJ level using a high speed diamond-coated bur under copious water coolant to obtain roots with 11 mm lengths. All roots were covered with a fine layer of silicon impression material simulating the periodontal ligament (PDL) and were then embedded in acrylic blocks. With a random number table the specimens were divided into four groups (*n*=25). In groups 1 to 3, specimens were prepared using Mtwo up to size 25/0.07, PTU up to F2 and RCP R25, respectively. In group 4, the teeth were left unprepared (control group). Each instrument was installed on a handpiece attached to a torque-controlled electric motor (VDW, VDW, Munich, Germany) and was used according to the corresponding manufacturers’ instructions for each system.

**Table 1:** Pattern of defects among different groups

**Groups**	**Specimens N (%)**		**Total**
	**Defected**	**No defect**	
**ProTaper**	6 (24%)	19 (76%)	25 (100%)
**Mtwo**	6 (24%)	19 (76%)	25 (100%)
**Reciproc**	1 (4%)	24 (96%)	25 (100%)
**Control**	0 (0%)	25 (100%)	25 (100%)

**Figure 1:**
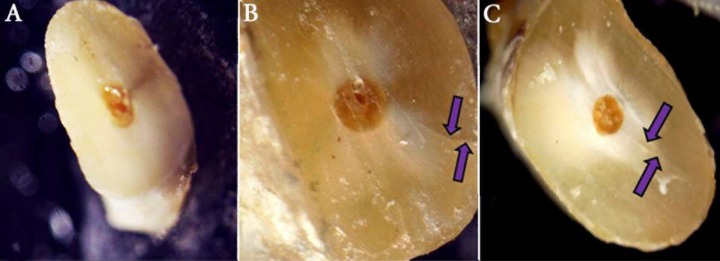
Patterns of fracture; *A)* No defect; no fracture line is visible, *B)* Fracture; complete fracture line (arrows) extending to the external root surface and *C)* Defected; incomplete fracture line (arrows) that did not reach the external root surface

Apical patency was established with a #15 K-File (Mani Inc., Togichi, Japan) prior to preparation of each canal. During preparation, 2.5% sodium hypochlorite (total volume of 12 mL per canal) was used as the irrigant. Prepared roots were finally rinsed with 2 mL of purified filtered water. All roots were sectioned at 3, 5 and 9-mm distances from the apex by a low speed saw (Leica, SP1600, Wetzlar, Germany) with water coolant. A total of 75 slices were obtained for each group to be blindly inspected for presence of cracks (under 12× magnification). According to Bürklein *et al.* [5], the fracture pattern was categorized as follows: *type I, no defect*; when no fracture was detected inside the root canal, *type II, fracture*; a complete fracture line starting from root canal wall to the root surface, *type III, defected*; an incomplete fracture line starting inside the root canal wall but did not reach the root surface. (Figure 1). The results were expressed as the number and percentage of cracked roots in each group.

The data was analyzed using the chi-square and Fisher’s exact tests and the level of significance was set at 0.05.

## Results

Table 1 shows defect patterns among different groups. Fracture category (*type II*) was not detected in any group. No defects were observed in control group. Six roots (24%) from Mtwo and PTU and only one root (4%) from RCP group showed dentinal crack formation. In total, crack formations were observed in 13 teeth from all experimental groups (17.3%). Based on fracture pattern, all these cracks were exclusively categorized as defected (*type I*). Regarding crack formation, there was no significant difference between RCP and control samples (*P*=0.300). Both Mtwo and PTU showed more cracks than RCP (*P*=0.042) and control *(P=*0.022*).* The difference between Mtwo and PTU was not significant (*P*=1.000).

## Discussion

This study evaluated dentinal crack formation following root canal preparation with RCP, PTU, and Mtwo. RCP showed less dentinal defects than PTU and Mtwo. No defect was shown in unprepared (control) specimens.

Rather than an instant phenomenon, VRF is a gradual progression of dentinal crazes [15, 16]. Mastication forces may progress these crazes into a complete VRF [1]. However, it is yet unclear whether craze lines and incomplete cracks may propagate into complete cracks and fractures after completion of the root canal treatment or not [5].

Rotary instruments produce significantly more dentinal defects than hand stainless steel instruments [17]. There is a direct relationship between excessive dentin removal and formation of root fractures [3, 14]; so highly tapered instruments make the root more prone to fractures [1, 17, 18]. 

According to the results of the present study, PTU and Mtwo systems produced more dentinal defects (24%) than RCP (4%) and control samples (0%); the incidence of instant root fracture was 0% among all experimental groups. This is similar to the results reported by Bier *et al.* [1] who reported cracks in 16% of the roots in mandibular premolars prepared with PTU. In contrast, Liu *et al.* [17] reported cracks in 50% of the roots instrumented with the PTU. However, Yoldas *et al.* [14] observed cracks in 30% of the mesial roots of mandibular teeth, that were instrumented with PTU. These contradictory results may be attributed to a number of reasons, and the most likely one is using teeth with different root canal anatomies [18].

Recent *in vitro* studies have focused on two factors that might make treated root canals susceptible to fracture: taper and design of files. Arbab-Chirani *et al.* [19] reported that progressive taper of F1 in PTU system makes the file highly stiff. This criterion may be the cause of more dentinal defects created with this system in the present study. On the other hand, Mtwo files are three times more flexible than PTU F1. However, in this study both systems showed similar results; this might be due to the same degree of taper that was chosen in both systems.

Preparation technique and the cross-sectional design of the instruments may affect the formation of dentinal defects. In this study full sequence rotary systems, Mtwo and PTU, formed more dentinal defects than RCP as a single file system. This finding probably depends on the type of instrument movement. It is stated that reciprocating systems, such as RCP and WaveOne, caused craze formation significantly more than Mtwo and PTU systems in single-canaled mandibular incisors. They used R40 (40/0.06) RCP instrument. The different findings might be due to the difference in tooth type and size/taper of the used instrument, while R25 (25/0.08) was used in this study. Regarding the effect of file design on formation of dentinal defects, Kim *et al.* [2] reported that the instrument design may affect stress/strain concentration in apical region which can lead to dentinal defects. 

In this study, cross section of selected instruments was not identical; PTU has a convex triangular cross section that offers strong cutting ability, while Mtwo and RCP are S-shaped [19]. In spite of the same cross section design of two latter systems, the difference between them was statistically significant which means that not only the design but also other factors, such as type of motion, are influential in this matter. Although Burklein *et al*. [5] explained that more cutting edges of S-shaped RCP is responsible for more craze line formation, their results showed the same outcome with WaveOne that has a triangle design. That might lead to a less significant role of file design. Berruti *et al.* [20] suggested that single-file systems remove smaller proportions of dentin than PTU. They concluded that reciprocation motion removes less dentine from root canal walls, therefore it is safer. This kind of motion also reduces the incidence of instrument separation. One important factor for imitating the clinical conditions in laboratory environment is considering the soft tissues surrounding teeth. In the present study samples were mounted in acrylic resin for imitating PDL. Burklein *et al.* [21], did not mount their specimens. On the other hand, they did not simulate periodontal ligament that may alter stress distribution pattern generated during root canal preparation. Periodontal ligament has an undeniable role in controlling either functional or parafunctional forces on teeth. This could be a strong evidence to back up the difference in results of aforementioned studies Literature suggest that taper and shape of the files as influencing factors could play a significant role in eventual creation of dentinal fractures [1, 2, 14]. 

Finally, it is important to point out that this study was designed and performed in an *in vitro* environment. Currently, there is an evident lack of correlation between the results obtained in this type of studies and the clinical situations. External factors such as masticatory forces, oral environment and *etc.* cannot be imitated in laboratory conditions.

There are several promising advantages mentioned for RCP; such as time saving, being safe and less probable to cause root fracture or instrument fragmentation [22-24].

## Conclusion

Considering the limitations of this study, our results suggested that Reciproc produces less dentinal cracks during root canal preparation.
